# A swaying between successive pandemic waves and pandemic fatigue: Where does Jordan stand?

**DOI:** 10.1016/j.amsu.2021.102298

**Published:** 2021-04-16

**Authors:** Ala'a B. Al-Tammemi, Zeinab Tarhini, Amal Akour

**Affiliations:** Department of Family and Occupational Medicine, Faculty of Medicine, University of Debrecen, H-4032 Debrecen, Hungary; Doctoral School of Health Sciences, University of Debrecen, H-4032 Debrecen, Hungary; Faculty of Medicine, University of Limoges, 87025, Limoges, France; Department of Biopharmaceutics and Clinical Pharmacy, School of Pharmacy, The University of Jordan, 11942, Amman, Jordan; Department of Pharmacy, Faculty of Pharmacy, Al-Zaytoonah University of Jordan, 11733, Amman, Jordan

**Keywords:** Jordan, COVID-19 pandemic, Precautionary measures, Mitigation strategies, Pandemic waves, Government-public partnership

## Abstract

This article highlights the main aspects of Jordan's public health response in combating the COVID-19 pandemic. Also, it briefly describes the main characteristics of the pandemic waves. Although Jordan has successfully implemented various stringent control measures at the early stage of the pandemic which resulted in a slow pace of COVID-19 spread in the country, the dramatic and sudden surge in COVID-19 cases and deaths since September 2020 raises many concerns and questionable debates regarding the effectiveness of Jordan's COVID-19 mitigation strategies, the earlier epidemiological surveillance process, decision-making and decisions' execution at various sectors, as well as the degree of commitment to precautionary measures among the general population. Jordan has passed through three distinct pandemic stages so far, and each stage provides lessons that can be used to improve the national preparedness and response plan in the future. This pandemic has afflicted most life domains; thus, sharing the responsibility and efforts between the government and people in combating it, is expected to be more efficient and effective than a one-sided response. Pandemic fatigue can act as a major risk factor for losing such a battle. The people of Jordan have been already through an unforgettable 2020 year that impacted them physically, emotionally, and even financially. Therefore, reliable actions should be considered by the decision-makers to provide sufficient support for the society. Also, strengthening the government-public partnership is a cornerstone for a successful, solid, and effective public health response, especially in times of an exhaustive pandemic crisis like the COVID-19.

## Highlights

• The COVID-19 pandemic is one of the most devastating events that impacted various life domains globally.
• Jordan was amongst the first countries to implement stringent control measures, and this has resulted in a delayed first pandemic wave.
• Collaborative efforts between the government and the general public are crucial for a solid and effective public health response.
• Pandemic fatigue and vaccine hesitancy may be the most current challenging issues , and both of them may worsen the epidemiological situation.

## 1 Overview of the COVID-19 pandemic

It has been over a year since the initial outbreak of Coronavirus Disease – 2019 (COVID-19). With a rapid and extensive spread, the COVID-19 pandemic has already attacked 223 countries and territories resulting in more than 113 million confirmed cases while the death toll surpassed 2.5 million deaths globally, as of February 28, 2021 [1]. Like many countries, Jordan, a country located in the Eastern Mediterranean Region (EMR) was not an exception to being severely impacted by the pandemic. However, being a middle-income country with limited resources in an unstable zone of the Middle East has imposed additional challenges to fight the pandemic effectively and efficiently.

**Fig. 1:**
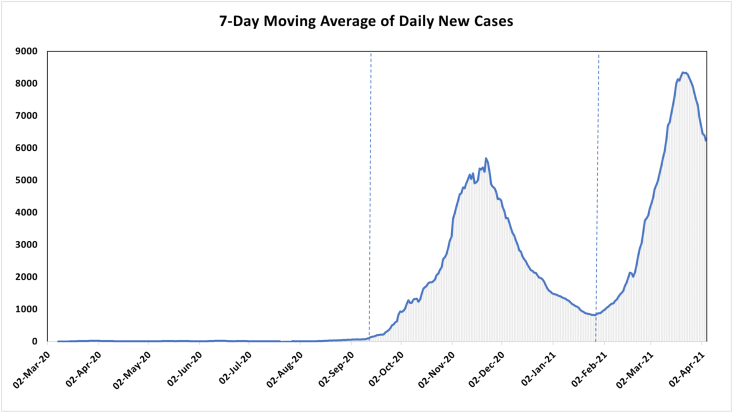
7-Day Moving Average of Daily New Cases of COVID-19 in Jordan During the period March 2, 2020–April 04, 2021. This figure was developed based on datasets retrieved from (corona.moh.gov.jo/en) [13] and Dong, E., Du, H., & Gardner, L. (2020) [26].

No one would deny that the Jordanian public health response has shown a promising and exemplary way of controlling the early stage of the pandemic [2]. The national response was initiated by implementing various stringent mitigation measures and non-pharmacological interventions entailing various life domains such as international travel, work duties at public and private sectors, educational sector, entertainment, religious and social events as well as health services delivery [2]. Also, modern digital channels were used to deliver numerous services, to interact with people, and in contact-tracing [2]. In line with other countries, a total lockdown with a nationwide curfew was also declared and enforced by laws [2]. Consequently, these initial measures have led to a slow pace of COVID-19 spread in the community which can be confirmed by the publicly declared statistics on the COVID-19 platform provided by the Jordanian Ministry of Health (corona.moh.gov.jo/en). Since March 2, 2020, which was the date of the first reported case of COVID-19 in Jordan, and as of April 04, 2021, the epidemiological trend has posed three distinct stages so far (See Fig. 1). The following sections will briefly describe the main characteristics of the three stages of the COVID-19 pandemic in the country. The dates of waves represent an approximate gross estimation.

## 2 Early stage of the pandemic in Jordan: a flat trend

The early pandemic stage in Jordan falls into the period between *March 2, 2020, and August 31, 2020*. In this period, the cumulative number of confirmed cases was 2034 with a total of 15 deaths. The epidemic curve during this stage was almost flat with very low daily cases. This period reflects the pandemic stage with the strongest control measures imposed by the government. The lockdown and curfew, public commitment to the COVID-19 precautionary behaviors, and law enforcement under the national defense orders were all contributing factors to sustain a slow, somehow unnoticed, pandemic existence in the country [2,3]. This stage was also characterized by the easing up of national restrictions that was started in early June 2020, aiming for a gradual return to a full-functioning economy and post-pandemic normality in Jordan. The general population, healthcare workers, and students were also psychologically impacted by the pandemic during this stage, and this has been addressed in various literature [[4], [5], [6], [7]].

The healthcare system in Jordan was already suffering from many challenges prior to the current pandemic, and these challenges were significantly increased during the COVID-19 crisis [8]. However, there was no ground of evidence that the government or decision-makers have effectively utilized the early period of the pandemic control (March–August 2020) to enhance the capacity of the Jordanian healthcare system, especially Intensive Care Unit (ICU) beds, ventilators as well as the health workforce, and this could have serious impacts during the real pandemic waves. The Jordanian decision-makers should have considered that alongside the COVID-19 patients, there is/will be a large number of patients with various illnesses and therapeutic needs, imposing an inherent responsibility to properly use the financial resources received from various national and international funding authorities during the state-of-control over the first pandemic stage to manage the health system capacity issue [[9], [10], [11], [12]].

## 3 The first COVID-19 pandemic wave: the real crisis

Unexpectedly, the COVID-19 cases and deaths have dramatically escalated since September 2020, when Jordan started to register daily cases in hundreds and later in thousands. This was a turning point in the epidemiological status of the country; the first pandemic wave has just begun. This approximately relates to the period spanned from *September 1, 2020, until January 26, 2021*. Unfortunately, the first wave has resulted in about 320,207 new cases and 4233 deaths [13]. The unprecedented change in the epidemiological situation in Jordan since early September 2020 raises many concerns and questionable debates regarding the effectiveness of Jordan's COVID-19 mitigation strategies, the earlier epidemiological surveillance process, the decision-making procedures and their implementation at various sectors and levels, as well as the degree of commitment to the preventative measures at the individual, community, and institutional levels.

During the same period stated above, many countries globally were experiencing a surge in COVID-19 cases and deaths, however, most of these countries were also hit severely during the early pandemic stage, unlike Jordan. Nevertheless, several possible scenarios might be behind the significant rise in COVID-19 cases and deaths which is noticeable from September 2020 in Jordan. Some of which are **(i)** The frequent changes in the decision-making procedure which resulted in reluctant decisions such as the Friday-Saturday nationwide curfew which was after few days changed to only-Friday nationwide curfew [14]. This has created a state of confusion in the Jordanian society, questioning the epidemiological rationale behind such decisions and afflicting the public-government trusteeship. Such a decision (frequent on-off curfew policy) was associated with distress and chaos around weekends and during weekdays, leading to overcrowding at certain times of the day. Government officials claimed that Friday's nationwide curfew policy aims at reducing social gatherings which are traditionally used to occur on Fridays and to provide a better opportunity for the contact-tracing process. However, and upon declaring this policy, the people of Jordan started to hold their social gatherings on other days as observed on social media platforms from people's public posts and comments. Besides, the on-off curfew policy with rapid turn-over in decisions regarding public and private labor sectors are expected to negatively impact the psychological well-being of people, especially with the feeling of uncertainty about their future, and their concerns about the economic impacts of these decisions, considering the lack of sufficient financial support. To the best of our knowledge and apart from the government officials' statements regarding the effectiveness of the Friday round-the-clock curfew and the weekdays night partial curfew policy and its impact on the epidemiological trend, we could not retrieve a published scientific study that examined the effectiviness of this specific policy utilizing the country's own epidemiological data **(ii)** The initial low numbers of reported cases and deaths in Jordan might have given an unreal sense of relaxation and victory to both decision-makers and the general public which could impact the overall commitment to pandemic-related precautionary measures, **(iii)** The campaigns of the 2020 Jordanian parliamentary election, which were held by most candidates across the country during September–October 2020 with an almost no-to-minimal commitment to COVID-19 preventive measures and neglecting the dangerous situational updates in Jordan [15]. Later, these campaigns were banned by the independent election committee due to numerous violations of public health measures. Moreover, after declaring results, hundreds of illegal public celebrations were held by people across the country, celebrating the success of their electoral candidates and violating the defense law orders of post-election total curfew for few days [16], **(iv)** Dereliction in auditing the implementation of preventive and control measures at the individual, community and institutional levels across the country for few months and before more serious law-enforced measures were implemented recently, **(v)** Changes that could happen to the transmissibility, pathogenicity and virulency of SARS-CoV-2 in the EMR, and lastly **(vi)** The dwindling in following the recommended preventative measures among the general population, namely, physical distancing, using masks in crowded areas and practicing various hygienic measures.

However, it can be noticed from Fig. 1 that from late November 2020, the epidemiological curve of daily new cases started to decline, yet with a large number of new daily cases, and this could be due to an increased commitment to the precautionary measures among the general population, especially after law enforcement and when people started to realize the true picture of the situation that endangered many lives of their beloved ones.

Fortunately, during this pandemic wave, decision-makers were too willing to catch up with what they missed in the period of March–August 2020, and more serious steps were declared in late October 2020 to enhance the health system capacity by constructing field hospitals designated for COVID-19 cases, alongside recruiting healthcare workers to support the existing health workforce, creating some hope to improve the epidemiological situation in Jordan by learning it the hard way [17]. Besides, these epidemiological repercussions have called for the establishment of the National Center for Epidemics and Communicable Diseases Control to continue combating the current pandemic and to handle any future outbreaks in well-systematized, effective, and efficient ways [18].

## 4 The second COVID-19 pandemic wave: is it pandemic fatigue?

Despite the “wax and wane” in the Jordanian battle against the COVID-19 pandemic, the battle has not yet finished. A successive second pandemic wave has hit Jordan starting around *January 27, 2021,* and the COVID-19 morbidity and mortality were rising again (See Fig. 1). Since the start of this wave and as of April 04, 2021, around 310,666 new cases and 2953 deaths have been registered. This pandemic wave has posed significant burden on healthcare institutions with high occupancy rates of isolation beds, ventilators, and ICU beds, especially in northern and central Jordan. As of April 04, 2021, the occupancy rate of ICU beds due to COVID-19 was about 73% and 77% in northern and central regions, respectively [13]. The statistical figures were also worrying regarding the occupancy rate of ventilators which reached as high as 50% in some regions [13].

Despite starting the COVID-19 vaccine rollouts in the country [19], vaccine acceptance among the general population is still challenging with low rates of vaccine uptake [20]. Based on a recent study, around 28.4% of the Jordanian participants expressed their intention to take the COVID-19 vaccine [21]. The rapid development of COVID-19 vaccines might have led to a sense of fear and lack of confidence in vaccines safety which can result in vaccine hesitancy [[20], [21], [22]]. In addition, various factors could be behind this severe second wave, including failure to sufficiently adopting the recommended COVID-19 precautionary behaviors among the public and the evolving new variants of SARS-CoV-2. Besides, pandemic fatigue can also act as a major risk factor for such a deterioration in the epidemiological status in Jordan [19,23,24]. The people of Jordan have been already through an unforgettable 2020 year that impacted them physically, emotionally, and even financially. Therefore, reliable actions should be considered by the decision-makers to provide sufficient support for the Jordanian society, financially and psychologically.

## 5 The uncertainty of the COVID-19 battle in 2021: the hope for vaccine-accelerated herd immunity

As Jordan has already passed into an uncontrolled phase of community COVID-19 spread, there is now a significant need for refurbishing the government-public partnership for the best of the country. This pandemic has afflicted most of the human life domains; thus, sharing the responsibility and efforts between the government and people in combating it, is expected to be more efficient and effective than a one-sided response.

The recent approval of COVID-19 vaccines has been the beacon of hope for millions of people around the world to combat the pandemic more effectively through vaccine-induced herd immunity [25], but this global achievement could be perceived and handled by the general public in a wrong manner, impacting their commitment to COVID-19 precautionary measures. Accordingly, more nationwide efforts should be directed to raise awareness about vaccination, and about the importance of sustaining the adoption of physical distancing, wearing face masks, practicing respiratory etiquette, avoiding handshaking as well as avoiding crowded places. Also, the general population should take into consideration that universal and equitable access to vaccination cannot be guaranteed, especially in the early stages of arrival. However, people should consider taking the vaccines once available as this will facilitate reaching herd immunity. Besides, these efforts can be supported by theory-based health promotion campaigns that may enhance the COVID-19 vaccine acceptance in the society. It is also important to emphasize that the COVID-19 precautionary behaviors should be considered as a “new normal” lifestyle during the year 2021 as well.

On the other hand, Jordanian decision-makers of the COVID-19 task force are advised to provide the public with more evidence-based decisions coupled with reliable and sufficient economic and psychological support especially for those who have been severely afflicted by the economic impacts of the daily partial curfew policy and the Friday total curfew policy. Strengthening the government-public trusteeship is a cornerstone for a successful, solid, and effective public health response, especially in times of an exhaustive pandemic crisis like the COVID-19.

With a rapid turnover of decisions regarding the curfew policy (partial vs total), many business sectors, and the educational sector accompanied by many uncertainties about the long-term health and economic impacts of this crisis, all the previous issues could lead to a profound psychological impact and may deteriorate people's commitment to the imposed pandemic-related measures. Although the COVID-19 pandemic has forced most countries worldwide to be in an extraordinary and real crisis, even high-income ones, learning from other countries' experiences is vital to get a more comprehensive view to combat crisis by looking at the situation from different perspectives and through multiple lenses.

## 6 Conclusion

The COVID-19 crisis has forced Jordan to go through unprecedented changes and events that severely afflicted people's life. The successive pandemic waves, the agonizing rise in COVID-19 morbidity and mortality, the way of managing the early stage of the pandemic which was characterized by the negligence of the health system capacity issue, and has consequently impacted the national response during the real pandemic waves, the insufficient financial support provided to vulnerable people and to those who work in certain businesses, the shortage in COVID-19 vaccine supplies, and the lack of robust published epidemiological studies utilizing the country's own epidemiological data, all were factors that significantly impacted the pandemic preparedness and response in the country as well as many pandemic-related decisions. On the other hand, the people of Jordan have a paramount responsibility in adopting the COVID-19 precautionary measures and considering vaccination as these factors are considered one of the cornerstones for combating the pandemic in the current situation. Also, decision-makers and policymakers should consider the macro-and micro-socioeconomic influences in the national preparedness and response plan regarding the current pandemic and any future events. Determinants of pandemic fatigue should be carefully examined and addressed by appropriate actions to avoid any further deterioration in the epidemiological situation in Jordan.

## Provenance and peer review

Not commissioned, externally peer reviewed.

## Funding

This article did not receive any specific grant from funding agencies in the public, commercial, or not-for-profit sectors.

## Data availability

The datasets analyzed are publicly available and were cited in the figure's caption.

## Consent to participate

Not Applicable.

## Ethical approval

Not required.

## Declaration of competing interest

The authors have no conflicts of interest to declare that are relevant to the content of this article.
